# Reporting on FH-deficient renal cell carcinoma using circulating succinylated metabolites

**DOI:** 10.1172/JCI170195

**Published:** 2023-06-01

**Authors:** Divya Bezwada, James Brugarolas

**Affiliations:** 1 Children’s Medical Center Research Institute,; 2 Kidney Cancer Program, Simmons Comprehensive Cancer Center, and; 3 Department of Internal Medicine, Division of Hematology/Oncology, University of Texas Southwestern Medical Center, Dallas, Texas, USA.

## Abstract

Fumarate hydratase–deficient (FH-deficient) renal cell carcinoma (RCC) represents a particularly aggressive form of kidney cancer. FH-deficient RCC arises in the setting of germline, or solely somatic, mutations in the *FH* gene, a two-hit tumor suppressor gene. Early detection can be curative, but there are no biomarkers, and in the sporadic setting, establishing a diagnosis of FH-deficient RCC is challenging. In this issue of the *JCI*, Zheng, Zhu, and co-authors report untargeted plasma metabolomic analyses to identify putative biomarkers. They discovered two plasma metabolites directly linked to fumarate overproduction by tumor cells, succinyl-adenosine and succinic-cysteine, which correlate with tumor burden. The identification of circulating biomarkers of FH-deficient RCC may aid in the diagnosis of FH-deficient RCC and provide a means for longitudinal follow-up.

## Fumarate hydratase and tumorigenesis

Hereditary leiomyomatosis and renal cell carcinoma (HLRCC) is a rare familial cancer syndrome with an autosomal dominant pattern of inheritance characterized by germline mutations in the TCA cycle enzyme fumarate hydratase (FH). Patients with HLRCC develop cutaneous and uterine leiomyomas as well as FH-deficient renal cell carcinomas (RCCs). FH-deficient RCCs require diligent treatment, as even small primary tumors can give rise to regional and distant metastases (1). There are no specific FDA-approved therapies for metastatic FH-deficient RCC, and despite encouraging results in vitro, early attempts at targeting codependent pathways, such as glycolysis, did not show promise (2).

The gene encoding FH is a two-hit tumor suppressor gene inactivated in sporadic and familial, HLRCC-associated, RCC. At the cellular level, loss of FH causes accumulation of the substrate fumarate, which can reach millimolar concentrations inside the cell (3). At these high concentrations, fumarate acts as an oncometabolite that induces metabolic reprogramming and drives tumorigenesis. Specifically, fumarate induces hypermethylation caused by inhibition of histone and DNA demethylases, leading to epithelial-mesenchymal transition and *CDKN2A* inactivation (4, 5). In addition, fumarate activates HIF through HIF prolyl hydroxylase inhibition (6) and induces a nuclear factor erythroid 2–related factor 2–mediated (NRF2-mediated) antioxidant program through kelch-like ECH-associated protein 1 (KEAP1) inhibition (7).

At the molecular level, fumarate drives succinylation, a posttranslational modification that adds succinyl groups to proteins and peptides, including glutathione (8–10). The widespread metabolic reprogramming of FH loss has been studied in cells and tissues, but it is unclear whether FH loss in tumors causes systemic alterations that can be detected in the blood.

## Identifying candidate biomarkers of FH loss

In this issue of the *JCI*, Zheng, Zhu, and colleagues (11) close this knowledge gap by analyzing circulating plasma metabolites from patients with FH-deficient RCC. They identified two terminal metabolites, succinyl-adenosine (suc-ado) and succinic-cysteine (suc-cys), that were elevated in the plasma. These two metabolites distinguished patients with FH-deficient RCC from those with simply germline mutations in *FH*, as well as from patients with other types of RCC.

Zheng, Zhu, and co-authors performed untargeted mass spectrometry metabolomic analyses of human plasma from a testing cohort of patients with FH-deficient RCC (*n* = 10), sporadic RCC (clear cell RCC [ccRCC], papillary RCC, etc.) (*n* = 10), and healthy adults (*n* = 10). The researchers identified several circulating metabolites that correlated with FH-mutant RCC and tumor size. While fumarate was elevated in the plasma of patients with FH-mutant RCC, its elevation was not specific to FH-deficient RCC (11). Indeed, circulating plasma fumarate is highly variable in healthy adults, reflecting the possible influence of diet and polymorphisms affecting *FH* expression in the general population (12). In contrast, suc-ado and suc-cys levels correlated with FH-deficient RCC and tumor burden (11).

The authors compared the levels of these candidate biomarker metabolites in a second validation cohort of patients with FH-deficient RCC (*n* = 83), patients with sporadic RCC (*n* = 77), and healthy adults (*n* = 78). Suc-ado and suc-cys levels were elevated in the patients with FH-deficient RCC, in particular in those with stage III or IV tumors, by comparison with the other groups. Furthermore, blood levels of suc-ado and suc-cys in *FH* germline mutation carriers were indistinguishable from those in healthy adults, which is consistent with the notion that FH needs to be inactivated before the metabolites are overproduced (11).

Suc-ado and suc-cys levels were further evaluated using a cell line and patient-derived xenograft (PDX), which showed similar results. Suc-ado and suc-cys blood levels increased proportionally with the growth of an FH-deficient PDX in mice but not with a ccRCC PDX. In addition, suc-ado and suc-cys levels acutely fell after resection of the FH-deficient tumors (11).

## Tracking suc-ado and suc-cys

**Figure 1:**
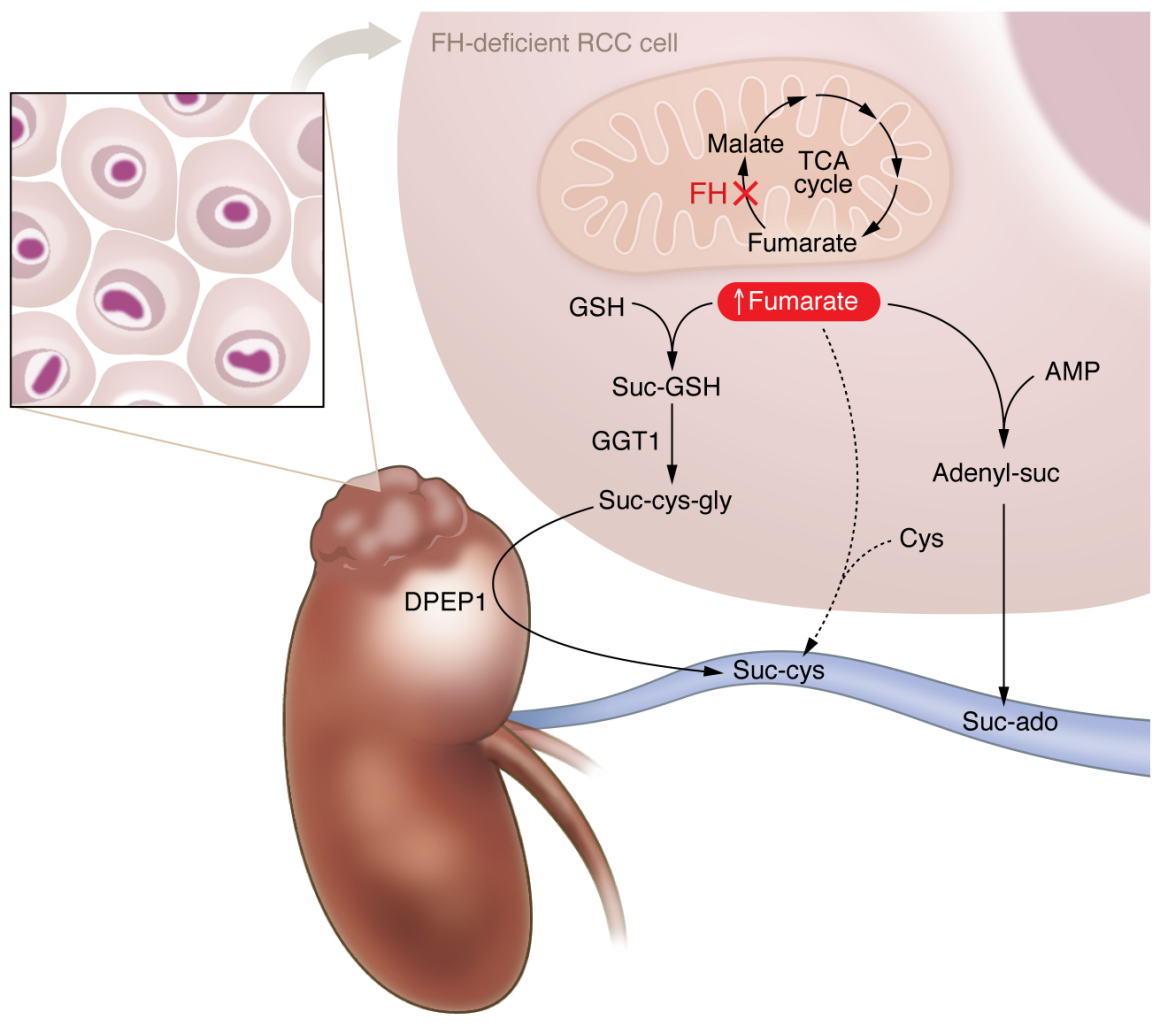
FH-deficient RCC produces suc-ado and suc-cys. Loss of the FH enzyme results in aberrantly high concentrations of fumarate in FH-deficient RCC. Increased levels of fumarate cause the accumulation of suc-GSH, which is converted to suc-cys-gly by the enzyme GGT1. Suc-cys-gly is released from the tumor and acted upon by a renal tubule DPEP1 enzyme that generates suc-cys. High concentrations of fumarate in tumor cells also cause the accumulation of adenyl-suc, leading to suc-ado. Both suc-cys and suc-ado are found in the blood stream, and their levels correlate with tumor mass.

To explore how suc-ado and suc-cys are linked to FH-deficient RCC, Zheng, Zhu, and colleagues investigated how they were produced (11). As previously reported, increased levels of fumarate led to increased levels of adenyl-suc and in turn increased production of suc-ado (6, 13). Prior work from the same group showed that succinic-glutathione (suc-GSH) accumulates in human and mouse FH-deficient tumor tissues, but suc-GSH is not detected in plasma. Suc-GSH is metabolized to succinic-cysteine-glycine (suc-cys-gly) by γ-glutamyltransferase 1 (GGT1) in tumors, and suc-cys-gly is further broken down by a renal tubular dipeptidase (DPEP1) to form suc-cys (Figure 1). As a result, the increased levels of both suc-cys and suc-ado in the circulating plasma of patients could be traced directly back to overproduction of fumarate by the tumors (11).

Finally, the authors tested the utility of these two metabolites for monitoring patients with FH-deficient RCC. Specifically, they followed five patients who developed metastatic disease and underwent a variety of interventions, both focal and systemic, over the course of several years. Suc-ado and suc-cys levels in the blood correlated with tumor burden, showing substantial reductions following surgery, and they tracked with the response to systemic therapy including targeted therapy and immunotherapy (11).

## Conclusions and future directions

The authors deserve commendation for focusing on an unmet medical need, their rational metabolomics approach, the mechanistic studies and assessment in mouse models, and their longitudinal analyses in a few patients. Additional studies will be required to assess the sensitivity and specificity as well as the clinical utility of suc-ado and suc-cys for the detection and management of FH-deficient RCC.

The data presented by Zheng, Zhu, and colleagues (11) suggest that suc-ado and suc-cys may be used to monitor treatment response and disease burden over time. Thus, these readily accessible blood biomarkers could complement routine imaging studies, and their utility deserves to be explored in prospective clinical trials.

Another setting in which circulating biomarkers of FH-deficient RCC would be useful is in the detection of subclinical disease. Biomarkers would be useful, for example, to ascertain whether there may be unappreciated subclinical disease after resection of an isolated primary tumor. However, the observation that suc-ado and suc-cys levels became undetectable in a patient who had an overt recurrence later on raises questions about the sensitivity of these metabolites to detect minimal residual disease. The same concern applies to the detection of undiagnosed FH-deficient RCC in patients with HLRCC.

Circulating biomarkers of FH-deficient RCC could also be helpful in the evaluation of small renal masses in the sporadic setting. Small renal masses are often followed with periodic surveillance scans to assess their growth over time, and biopsies are not always performed. However, if a tumor was determined to be FH-deficient, this would likely prompt early intervention. With the availability of reliable blood biomarkers, more customized approaches could be offered.

One particularly promising application is in the pathological diagnosis of sporadic FH-deficient RCC. Genetic testing is not routinely performed on sporadic RCC, and histological analyses are insufficient for diagnosis (14). The pathological diagnosis can be aided by IHC studies for FH and cysteine succination using an anti–S-(2-succino)-cysteine (anti-2SC) antibody. However, even with IHC studies, the diagnosis is not always straightforward (14, 15) and could be complemented by analyses of suc-ado and suc-cys levels in the blood.

Another question pertains to the specificity of suc-ado and suc-cys for FH-deficient RCC. Patients with germline *FH* mutations are also predisposed to uterine leyomyomas, which can reach a large size. How uterine leyomyomas would affect circulating levels of suc-ado and suc-cys is unclear from this study (11). Conversely, as the FH enzyme is a homotetramer, it is possible that some point mutants may exhibit dominant negative activity and substantially reduce FH activity in nontumor cells in patients with HLRCC. How this may impact suc-ado and suc-cys levels remains to be fully determined. In addition, the generation of suc-cys involves a renal tubular dipeptidase (DPEP1), and the impact of reduced renal mass (i.e., nephrectomy) and renal dysfunction on suc-cys levels remains unknown.

Moving forward, suc-ado and suc-cys should be evaluated in prospective trials before they are routinely incorporated in the clinic. Future studies should also address the added value of measuring both metabolites. The study by Zheng, Zhu, and colleagues (11) is a step in the right direction to identify clinically relevant biomarkers for a devastating subtype of kidney cancer.

## Version 1 06/01/2023

Electronic publication
